# Bupropion Use and Risk of Open-Angle Glaucoma among Enrollees in a Large U.S. Managed Care Network

**DOI:** 10.1371/journal.pone.0123682

**Published:** 2015-04-13

**Authors:** Joshua D. Stein, Nidhi Talwar, Jae H. Kang, Olivia I. Okereke, Janey L. Wiggs, Louis R. Pasquale

**Affiliations:** 1 Department of Ophthalmology and Visual Sciences, University of Michigan Medical School, Ann Arbor, Michigan, United States of America; 2 Department of Medicine, Channing Division of Network Medicine, Brigham and Women’s Hospital, Harvard Medical School, Boston, Massachusetts, United States of America; 3 Harvard School of Public Health, Harvard Medical School, Boston, Massachusetts, United States of America; 4 Department of Psychiatry, Brigham and Women’s Hospital and Harvard Medical School, Boston, Massachusetts, United States of America; 5 Department of Ophthalmology, Harvard Medical School, Boston, Massachusetts, United States of America; Casey Eye Institute, UNITED STATES

## Abstract

**Objective:**

Tumor Necrosis Factor (TNF) mediates retinal ganglion cell death in glaucoma. Anti-TNF drugs are neuroprotective in an animal model of glaucoma. It is unclear whether medications with anti-TNF properties such as bupropion have an impact on the risk of developing open-angle glaucoma (OAG) in humans. The purpose of this study is to determine whether bupropion use alters the risk of developing OAG.

**Methods:**

Claims data for beneficiaries age ≥35 years with no pre-existing OAG enrolled in a large nationwide U.S. managed care network continuously for ≥4 years between 2001-2011 was analyzed to identify patients who had been newly-diagnosed with OAG. The amount of bupropion use as captured from outpatient pharmacy claims over a four-year period was also quantified for each beneficiary. Multivariable Cox regression modeling assessed the impact of bupropion and other antidepressant medications on the risk of developing OAG with adjustment for sociodemographic characteristics of the enrollees along with medical and ocular comorbidities.

**Results:**

Of 638,481 eligible enrollees, 15,292 (2.4%) developed OAG. After adjustment for confounding factors including use of other antidepressant medication classes, each additional month of bupropion use was associated with a 0.6% reduced risk of OAG (HR = 0.994, (95% CI: 0.989-0.998), p = 0.007). Compared to nonusers, those with 24-48 months of bupropion use had a 21% reduced hazard (HR=0.79, (CI: 0.65-0.94), p = 0.0099) of OAG. This association did not differ among persons taking bupropion for depression or for other reasons (p-interaction = 0.82). There was no significant association between use of tricyclic antidepressants (HR = 1.000, (CI: 0.997-1.004), p = 0.95) or selective serotonin reuptake inhibitors (HR = 0.999, (CI: 0.997-1.001), p = 0.39) and development of OAG.

**Conclusion:**

These findings suggest bupropion use may be beneficial in reducing the risk of OAG. If prospective studies confirm the findings of this analysis, this may identify a novel therapeutic target for OAG.

## Introduction

Randomized clinical trials (RCT) suggest that intraocular pressure (IOP)-lowering medications, laser trabeculoplasty, or glaucoma filtration surgery slow disease progression for patients with open-angle glaucoma (OAG) but these measures are not curative.[1–3] Importantly, some patients with OAG appear to demonstrate disease progression despite achieving low IOPs, suggesting that non-IOP related factors might contribute to or perpetuate optic nerve damage. The discovery, characterization, and identification of effective treatments of risk factors for OAG besides lowering IOP can reduce the burden of vision loss from this disease.

In animal models [4,5] and in humans,[6–8] elevated IOP serves as a stressor to incite neuro-inflammation with activation of immune cells resident to the retina and optic nerve. These immune cells produce cytokines such as tumor necrosis factor (TNF) that bind to TNF receptor 1, which is also upregulated in the human glaucomatous optic nerve.[6,8,9] Intravitreal TNF injection mimics glaucomatous damage in normal mice; furthermore, TNF binding to a related receptor, TNF receptor 2, mediates retinal ganglion cell death in a murine angle closure glaucoma model.[10] Intraperitoneal injection of enteracept, a medication which blocks TNF activity, has been found to be neuroprotective in a rodent model of glaucoma.[11] Ocular x-ray radiation prevents monocyte entry into the optic nerve and attenuates optic nerve damage in a murine model of glaucoma.[12] These latter data further support the notion that blocking neuro-inflammation may protect the optic nerve from glaucomatous damage.

Bupropion is an antidepressant that is also effective for aiding with smoking cessation.[13] It is a norepinephrine—dopamine reuptake inhibitor but exhibits less acetylcholine receptor antagonism and cardiac depressive activity than tricyclic antidepressants (TCAs).[14] The norepinephrine-dopamine reuptake inhibitory property is thought to also suppress TNF production. In fact, anecdotal reports suggest that bupropion can induce remission of recalcitrant Crohn disease [15] and atoptic dermatitis[16], two conditions known to be mediated by TNF.[17,18] Furthermore, bupropion delivered via gastric lavage ameliorated intestinal damage and reduced serum TNF levels in a rodent model of intestinal ischemia-reperfusion injury.[19] Finally, orally administered bupropion blocked depression-like symptoms produced when TNF was injected into the ventricular space of the mouse brain.[20] These investigations offer evidence that bupropion activity may include targeting TNF production secondary to neuro-inflammatory processes. Since studies suggest that neuro-inflammation plays a role in animal models of glaucoma[21], and there is mounting evidence that bupropion may help reduce neuro-inflammation, we assessed the relationship between bupropion use and the risk of OAG among a large cohort of patients enrolled in a managed care network throughout the United States to determine whether use of this medication affects the risk of developing OAG.

## Methods

### Data Source

The Clinformatics DataMart database (OptumInsight, Eden Prairie, MN) contains health care claims data for all beneficiaries in a managed-care network with enrollees throughout the United States. The dataset we used comprises all enrollees with ≥1 International Classification of Diseases, Ninth Revision-Clinical Modification (ICD-9-CM) code for an eye-related diagnosis (360–379.9); ≥1 Current Procedural Terminology (CPT) code for any eye-related visits, or diagnostic or therapeutic procedures (65091–68899 or 92002–92499) from January 1, 2001, through December 31, 2011. We had information on all these enrollees’ medical claims for ocular and nonocular conditions; sociodemographic information, including age, sex, race, education level and income; and outpatient pharmacy prescriptions that were filled. Beneficiaries in the medical plan were also fully enrolled in the pharmacy plan. We have previously used this database to study the relation between various exposures and OAG.[22–24] Because the data are de-identified, the University of Michigan Institutional Review Board approved this study as a non-regulated study.

### Participants and Sample Selection

Patients were included in the analysis if they met these criteria: age ≥35 years, continuous enrollment in the medical plan for ≥4 years with ≥2 visits to an eye-care provider (ophthalmologist or optometrist). Individuals with pre-existing OAG (≥1 diagnosis during a 4-year “look-back” period) were excluded, as were those with fewer than 4 years in the plan, those with non-continuous enrollment, and those younger than 35 years of age at the time of plan enrollment. Previous work indicated that implementing a 4-year “look back” period enables researchers to distinguish incident from non-incident cases of OAG in claims data.[25]

### Bupropion Use

Individuals were classified as receiving bupropion if they had ≥1 outpatient pharmacy prescription for this medication as identified based on American Hospital Formulary Service drug codes. The database contains information on the number of days for which an enrollee was supplied a given medication, thereby enabling us to quantify the amount each beneficiary was prescribed during their time in the plan.

### Dependent Variable

The development of OAG was the dependent variable for this analysis. Beneficiaries with OAG were identified by ICD-9-CM codes 365.1, 365.10, 365.11, 365.12, and 365.15. Beneficiaries were identified as developing incident OAG if they received no diagnosis of OAG during the 4-year look-back period and then went on to receive a diagnosis of OAG during their subsequent time in the plan. A recent study showed that billing codes are >90% accurate in identifying patients with OAG, as confirmed with chart review.[26]

### Analyses

We used SAS software, version 9.3 (SAS Institute, Cary, NC) to perform all statistical analyses. Participant characteristics were summarized for the sample by using means and standard deviations for continuous variables and frequencies and percentages for categorical variables.

Cox proportional hazards regression with delayed entry was used to estimate the hazard for developing OAG associated with bupropion use. We used the first 4 years of beneficiaries’ enrollment in the plan as their look back period, after which the index date marked the beginning of the follow-up period. Individuals with ≥1 diagnosis of OAG during the look-back period were considered non-incident cases of OAG and were excluded from the analysis. To ensure that each beneficiary had an opportunity to receive a diagnosis of OAG in the look-back period, we required all included beneficiaries to have ≥1 visit to an eye-care provider during this time. We also required a visit to an eye-care provider during the follow-up period, to allow for an opportunity to receive a diagnosis of OAG during that interval. Beneficiaries were followed in the model from the index date until they developed OAG or were censored. Censoring occurred at the last visit to an eye care provider. The key predictor variable in our models was bupropion use, which was treated as a time-dependent covariate. The number of days each beneficiary was covered by a bupropion prescription was totaled over a 4-year moving time window enabling quantification of medication exposure from the index date to the date of OAG onset or censoring. The look-back period ensured that enrollees had a 4-year window available to quantify prior bupropion exposure. For the Cox regression model, we adjusted for the following covariates: age at index date, sex, race, income, region of residence at medical plan enrollment, urban/rural residence; the ocular comorbidities of cataract, pseudophakia or aphakia, proliferative and nonproliferative diabetic retinopathy, retinal vascular occlusion; and the nonocular comorbidities of diabetes mellitus, systemic hypertension, systemic hypotension, sleep apnea, cardiovascular disease, osteoporosis, and depression. The specific ICD-9-CM codes used to capture these conditions can be found in **Table 1**. The final multivariable model included these predictors, along with bupropion use, selective serotonin reuptake inhibitor (SSRI) use, and tricyclic antidepressant (TCA) use. Another regression model was performed to assess whether there was a dose-response association between bupropion use and OAG. In this model we determined whether beneficiaries receiving more bupropion had a difference in risk of OAG compared to those with less exposure. In a third regression model, several interactions were considered to assess whether the risk of OAG differed among bupropion users versus nonusers by age, sex, race, or indication for its use. Checks of the models for multicollinearity were performed, and no significant multicollinearity was observed (all variance inflation factors < 3). For all analyses, p<0.05 was considered statistically significant.

**Table 1:** International classification of diseases, ninth revision, clinical modification (ICD-9-CM) codes used in the analysis.

Condition	ICD-9-CM Codes
Cataract	366, 366.0, 366.00, 366.01, 366.02, 366.03, 366.04, 366.09, 366.1, 366.10, 366.12, 366.13, 366.14, 366.15, 366.16, 366.17, 366.18, 366.19, 366.41, 366.45
Diabetes mellitus	250.0, 250.00, 250.01, 250.02, 250.03, 250.1, 250.10, 250.11, 250.12, 250.13, 250.2, 250.20, 250.21, 250.22, 250.23, 250.3, 250.30, 250.31, 250.32, 250.33, 250.4, 250.40, 250.41, 250.42, 250.43, 250.5, 250.50, 250.51, 250.52, 250.53, 250.6, 250.60, 250.61, 250.62, 250.63, 250.7, 250.70, 250.71, 250.72, 250.73, 250.8, 250.80, 250.81, 250.82, 250.83, 250.9, 250.90, 250.91, 250.92, 250.93, 362.01, 362.02, 362.03, 362.04, 362.05, 362.06, 362.07
Non-proliferative Diabetic retinopathy	362.01, 362.03, 362.04, 362.05, 362.06
Proliferative Diabetic Retinopathy	362.02
Myocardial infarction / Cardiovascular disease	410, 410.0, 410.00, 410.01, 410.02, 410.1, 410.10, 410.11, 410.12, 410.2, 410.20, 410.21, 410.22, 410.3, 410.30, 410.31, 410.32, 410.4, 410.40, 410.41, 410.42, 410.5, 410.50, 410.51, 410.52, 410.6, 410.60, 410.61, 410.62, 410.7, 410.70, 410.71, 410.72, 410.8, 410.80, 410.81, 410.82, 410.9, 410.90, 410.91, 410.92, 412
Hypertension	401, 401.0, 401.1, 401.9, 405, 405.0, 405.1, 405.01, 405.09, 405.11, 405.19, 405.9, 405.91, 405.99, 362.11, 402, 402.0, 402.00, 402.01, 402.1, 402.10, 402.11, 402.9, 402.90, 402.91, 403, 403.0, 403.00, 403.01, 403.1, 403.10, 403.11, 403.9, 403.90, 403.91, 404.0, 404.00, 404.01, 404.02, 404.03, 404.1, 404.10, 404.11, 404.12, 404.13, 404.9, 404.90, 404.91, 404.92, 404.93
Depression	296, 296.0, 296.00, 296.01, 296.02, 296.03, 296.04, 296.05, 296.06, 296.1, 296.10, 29611, 296.12, 296.13, 296.14, 296.15, 296.16, 296.2, 296.20, 296.21, 296.22, 296.23, 296.24, 296.25, 296.26, 296.3, 296.30, 296.31, 296.32, 296.33, 296.34, 296.35, 296.36, 296.4, 296.40, 296.41, 296.42, 296.43, 296.44, 296.45, 296.46, 296.5, 296.50, 296.51, 296.52, 296.53, 296.54, 296.55, 296.56, 296.6, 296.60, 296.61, 296.62, 296.63, 296.64, 296.65, 296.66, 296.7, 296.70, 296.71, 296.72, 296.73, 296.74, 296.75, 296.76, 296.8, 296.80, 296.81, 296.82, 296.89, 296.9, 296.90, 296.99
Hypotension	458, 458.0, 458.1, 458.2, 458.21, 458.29, 458.8, 458.9
Retinal Vein Occlusion	362.30, 362.31, 362.32, 362.35, 362.36
Open-angle glaucoma	365.1, 365.10, 365.11, 365.12, 365.15
Pseudophakia or aphakia	V43.1, 379.3, 379.31
Sleep apnea syndrome	327.2, 327.20, 327.21, 327.23, 327.27, 327.29, 780.51, 780.53, 780.57
Osteoporosis	733.00, 733.01, 733.02, 733.03, 733.09

## Results

**Fig 1:**
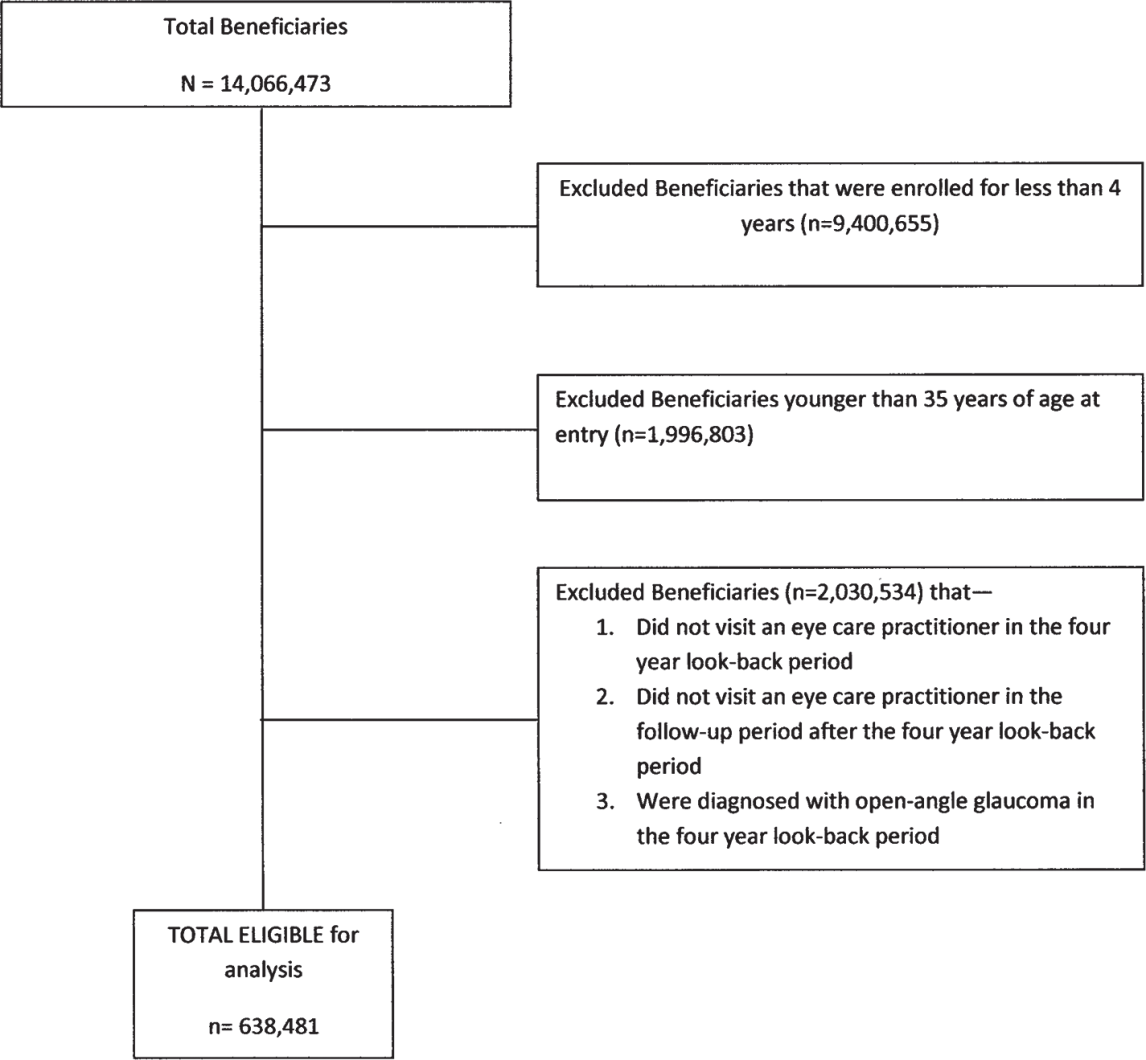
Sample selection process. Sample selection process for identifying elgible enrollees for the analysis.

There were 638,481 enrollees who met the study inclusion criteria (**Fig 1**). Beneficiaries who developed OAG were in the plan longer than those who did not develop OAG (mean ± SD: 7.5 ± 2.0 years versus 6.9 ± 1.9 years). (**Table 2**) Persons who developed OAG were older (mean age ± SD: 62.2 ± 11.7 years) than those who did not develop OAG (58.8 ± 12.0 years), and they were predominately of Caucasian ancestry. Most enrollees were educated at the high school level or higher and were earning $30,000 or more per year. (**Table 2**)

**Table 2:** Demographic characteristics.

	Enrollees who did not develop OAG	Enrollees who developed OAG
Study population, n (%)	623,189 (97.6)	15,292 (2.4)
Age at index date (years), Mean ± SD	58.8 ± 12.0	62.2 ± 11.7
Years in plan, Mean ± SD	6.9 ± 1.9	7.5 ± 2.0
Bupropion use n (%)	44,504 (7.1)	833 (5.5)
SSRI use n (%)	139,764 (22.4)	3,001 (19.6)
TCA use n (%)	49,134 (7.9)	1,178 (7.7)
Sex, n (%)		
Male	251,729 (40.4)	6,672 (43.6)
Female	371,460 (59.6)	8,620 (56.4)
Race, n (%)		
White	499,819 (80.2)	11,573 (75.7)
Black	21,457 (3.4)	1,037 (6.8)
Latino	28,326 (4.6)	892 (5.8)
Asian American	12,696 (2.0)	462 (3.0)
Other	5,147 (0.8)	124 (0.8)
Education*, n (%)		
< High school	5,814 (0.9)	216 (1.4)
High school diploma	186,241 (29.9)	4,840 (31.7)
Some college	240,693 (38.6)	5,790 (37.9)
College graduate	171,867 (27.6)	3,981 (26.0)
Advanced degree	1,774 (0.3)	33 (0.2)
Income*, n (%)		
<$30K	45,757 (7.3)	1,498 (9.8)
$30K-<$60K	184,638 (29.6)	4,855 (31.8)
$60K-<$100K	207,311 (33.3)	4,713 (30.8)
$100K-<$125K	78,540 (12.6)	1,717 (11.2)
≥$125K	69,953 (11.2)	1,650 (10.8)

OAG, Open-angle glaucoma; SSRI, selective serotonin reuptake inhibitor; TCA, tricyclic antidepressant; SD = standard deviation.

*There were 37,849 (5.9%) patients missing income, and 17,232 (2.7%) patients missing education.

### Bupropion Use

7.1% of the enrollees who met eligibility criteria (45,337 persons) had received ≥1 prescription for bupropion during their time in the plan. Among those taking bupropion, the mean ± SD days they took this medication was 466 ± 583 days. Of enrollees who had prescriptions for bupropion, 28,374 (62.6%) took it for >0–12 months, 6,244 (13.8%) took it for >12–24 months, 3,846 (8.5%) took it for >24–36 months, and 6,873 (15.2%) took it for >36 months. During follow-up, the median numbers of visits to eye care providers for bupropion users and nonusers was the same—5 visits each (p = 0.34).

### Development of OAG

Overall, 15,292 individuals (2.4%) received a diagnosis of incident OAG during their time in the medical plan. Relative to whites, blacks (adjusted HR = 2.08; 95% confidence interval (CI): 1.94–2.22, p<0.0001), Latinos (adjusted HR = 1.42; 95% CI: 1.32–1.53; p<0.0001), and Asian Americans (adjusted HR = 1.63; 95% CI: 1.48–1.80; p<0.0001) had an increased risk of developing OAG. Relative to beneficiaries with an income <$30,000, beneficiaries with an income of $100,000–125,000 or >$125,000 had a decreased hazard of developing OAG (adjusted HR = 0.81; 95% CI: 0.75–0.87; p<0.0001 and adjusted HR = 0.80; 95% CI: 0.74–0.87, p<0.0001, respectively) (**Table 3**).

**Table 3:** Multivariable analysis: risk factors for open-angle glaucoma^*^.

Risk Factor	Adjusted Hazard Ratio [95% Confidence Interval]	P-Value
Race^^^		
Black	2.08 [1.94–2.22]	<0.0001
Latino	1.42 [1.32–1.53]	<0.0001
Asian-American	1.63 [1.48–1.80]	<0.0001
Other	1.10 [0.92–1.32]	0.30
Income^†^		
$30K-$60K	0.95 [0.89–1.01]	0.08
$60K-$100K	0.87 [0.82–0.93]	<0.0001
$100K-$125K	0.81 [0.75–0.87]	<0.0001
>$125K	0.80 [0.74–0.87]	<0.0001
Medication Use^♦^		
Bupropion	0.994 [0.989–0.998]	0.007
SSRI	0.999 [0.997–1.001]	0.39
TCA	1.000 [0.997–1.004]	0.95

^*^ Multivariable regression analysis controlled for the following variables: age, sex, race, household income, region of residence, urban or rural residence, osteoporosis, retinal vascular occlusion, sleep apnea, depression, diabetes mellitus, hypertension, myocardial infarction, cataract, hypotension, non-proliferative and proliferative diabetic retinopathy, pseudophakia/aphakia, and an interaction between sex and depression.

^^^ Reference group: non-Hispanic whites.

^†^ Reference group: <$30,000.

^♦^ risk reduction for every additional 1 month of medication consumption. For example, every additional 1 month use of bupropion is associated with a 0.6% reduced hazard of developing open-angle glaucoma.

HR, Hazard Ratio; CI, Confidence Interval; OAG, Open-Angle Glaucoma; SSRI, selective serotonin reuptake inhibitor; TCA, tricyclic antidepressant.

### Bupropion Use and Open-Angle Glaucoma

The percentage of bupropion users who developed OAG was 1.8%. By comparison, 2.4% who had no record of bupropion use developed OAG (p<0.0001). In the multivariable analysis, after adjustment for age, other sociodemographic factors, ocular and systemic comorbidities, we identified a significant association between bupropion use and OAG. Every additional month of bupropion use reduced the hazard of OAG by 0.6% (adjusted HR = 0.994; 95% CI: 0.989–0.998; p = 0.007). We found no significant association between SSRI use (adjusted HR = 0.999; 95% CI: 0.997–1.001; p = 0.39) or TCA use (adjusted HR = 1.000; 95% CI: 0.997–1.004, p = 0.95) and OAG (**Table 3).**

### Dose-Response Association of Bupropion and Glaucoma

In another regression model where we categorized bupropion use into categories of use for different lengths of time, there was no statistically significant different risk of OAG among those with less than 24 months of bupropion use and those with no record of bupropion use (**Table 4**). By comparison, those with 24–48 months of bupropion use had a 21% decreased OAG risk (adjusted HR = 0.79, 95% CI: 0.65–0.94) relative to non-users of bupropion, a finding that was statistically significant (p = 0.0099).

**Table 4:** Hazard of developing open-angle glaucoma as a function of duration of bupropion use.

Duration of use (months)	Hazard Ratio (95% confidence interval)
Non-users	1.0 (REFERENCE)
>0–12	0.93 (0.83–1.03)
>12–24	0.91 (0.74–1.12)
>24–48	0.79 (0.65–0.94)

Controlled for selective serotonin reuptake inhibitors, tricyclic antidepressant use, age, sex, race, income, region of residence, urban/rural residence, medical diseases (hypertension, hypotension, myocardial infarction, diabetes mellitus, sleep apnea, osteoarthritis, depression), ocular diseases (cataract extraction, diabetic retinopathy, retinal venous occlusive disease), and an interaction between sex and depression.

### Interaction Model

In a separate regression model, several interactions were considered to assess whether the risk of OAG differed among bupropion users versus nonusers by age, sex, race, or indication for its use. After adjustment for the same covariates described above, the association between bupropion use and the risk of OAG was similar among enrollees <57 years old (the median age of the sample) and those age ≥57 years (p interaction = 0.99), among males and females (p interaction = 0.10), among non-Hispanic whites and persons of other races (p interaction = 0.63), and among persons with and without depression (p interaction = 0.82).

## Discussion

In this cohort of over 600,000 persons from throughout the United States, after adjusting for potential confounding factors, bupropion users had a significantly reduced hazard of developing OAG compared with non-users. Moreover, bupropion use for 24–48 months was associated with a 21% reduced hazard of OAG relative to no use and the protective effect associated with bupropion use was not observed with SSRIs and TCAs.

TNF is a biomarker associated with RGC death in patients with glaucoma.[9] Meta-analysis of six studies showed that aqueous humor levels of TNF were higher in persons with OAG than in control samples.[27] While no prior study has shown definitively that bupropion reduces TNF levels in humans, Brustolim and coworkers postulated that this drug decreases TNF synthesis by increasing extracellular norepinephrine and dopamine.[28] The secondary beta-adrenergic and D1 dopaminergic stimulation increases intracellular cyclic adenosine monophosphate (cAMP) and suppresses TNF synthesis. In a mouse model of endotoxemic shock induced by lipopolysaccharide, intraperitoneal bupropion reduced TNF levels and enhanced survival. These effects were ameliorated by antagonizing beta adrenergic and D1 receptors.[28] Suppression of TNF levels may serve as the biological basis for the inverse relation between bupropion use and OAG we are observing. Additional research using animal models of glaucoma are needed to substantiate our study’s findings and to understand the mechanism that might mediate such an effect.

Bupropion is a drug with complex pharmacological properties and the apparent inverse relation between bupropion use and OAG may not be related to suppressing TNF production; rather, it could be the result of a more direct effect of the drug on norepinephrine or dopamine metabolism. Moreover, the subsequent secondary effect of increasing intracellular cAMP could be beneficial in glaucoma, as cAMP signaling is important for both trabecular meshwork [29] and retinal ganglion cell function.[30] Yet, other antidepressants such as SSRIs and TCAs also increase cAMP levels and these agents did not appear to impact OAG risk in our analyses.

Bupropion has a fairly favorable side-effect profile.[31] Dry mouth and insomnia represent common side effects associated with bupropion use. Morning dosing can mitigate the latter effect. Other less common side effects are anxiety, nausea and headache. Since bupropion can increase the chance of seizure, it is contraindicated in epileptic disorders. Bilateral secondary angle closure is a potentially vision-threatening, but extremely rare side effect of bupropion use as there is only a single case report in the literature.[32]

This study has several limitations that need to be considered. The exposure of bupropion use was reported in terms of time receiving the medication and not in cumulative drug quantity consumed. Furthermore, we had no way to account for adherence to bupropion use, though non-adherence would likely drive our study results to the null. Also the precise indications for bupropion use in this analysis are not known. OAG was determined based on ICD-9-CM codes and not based on clinical data from actual medical records; however work by Muir and coworkers[26] reported excellent agreement between billing and medical records for this condition. Furthermore, misclassification of OAG status would also likely drive our results to the null unless differential misclassification by bupropion use occurred and this seems unlikely. Our interaction analyses suggest that no confounding by indication is operative in this analysis but we do not have data on which enrollees were current or former smokers. Previous work does not suggest that cigarette smoking is related to OAG,[33] although one study suggested that smoking was associated with increased risk of early onset glaucoma among African Americans.[34] Residual confounding is further minimized as known correlates of smoking (e.g., cardiovascular disease, macular degeneration) have been adjusted for. Finally, these study findings may not be generalizable to those with other forms of health insurance or patients without health insurance.

Strengths of this study include its large sample size with power to detect modest but potentially clinically relevant effects. Another strength is our ability to adjust for an array of potential confounding factors, although the extent that psychiatric co-morbidity is adjusted for in this analysis is not known. Bupropion use, as well as use of SSRIs and TCAs, relied on pharmacy data rather than patient self-report. We employed a look back period to remove persons with pre-existing OAG and prior studies suggest this is an accurate approach for capturing patients with newly diagnosed OAG.[25]

### Conclusions

This large study found that bupropion use might favorably alter the risk for OAG. More research is needed using animal models to determine whether bupropion is neuroprotective and whether this effect can be observed before damage to the optic nerve occurs. Future studies can also assess whether any protective effect is specific to bupropion or if the putative effect may extends to other FDA-approved drugs that alter TNF. Discovery of factors that might perpetuate progressive glaucomatous damage and of ways to reduce such damage could translate into reduced morbidity from this sight-threatening disease.
